# Differences in the Use and Opinions About New eHealth Technologies Among Patients With Psychosis: Structured Questionnaire

**DOI:** 10.2196/mental.9950

**Published:** 2018-07-25

**Authors:** Lucia Bonet, Blanca Llácer, Miguel Hernandez-Viadel, David Arce, Ignacio Blanquer, Carlos Cañete, Maria Escartí, Ana M González-Pinto, Julio Sanjuán

**Affiliations:** ^1^ Department of Clinic Medicine School of Medicine University of Valencia Valencia Spain; ^2^ Centre of Biomedical Investigation in Mental Health (CIBERSAM) Spanish Government Carlos III Health Institute Valencia Spain; ^3^ Department of Mental Health Sanitary Research Institute of Valencia (INCLIVA) Hospital Clínico of Valencia Valencia Spain; ^4^ Institute of Instrumentation for Molecular Imaging (I3M) Joint centre CSIC & Universitat Politècnica de València Valencia Spain; ^5^ Department of Mental Health Font of Sant Lluís Primary Care Centre Valencia Spain; ^6^ Centre of Biomedical Investigation in Mental Health (CIBERSAM) Spanish Government Carlos III Health Institute Vitoria Spain; ^7^ Department of Psychiatry BioAraba Research Institute Araba University Hospital, University of the Basque Country (EHU/UPV) Vitoria Spain

**Keywords:** eHealth, internet, mobile phone, viability, acceptability, psychosis, schizophrenia

## Abstract

**Background:**

Despite a growing interest in the use of technology in order to support the treatment of psychotic disorders, limited knowledge exists about the viability and acceptability of these eHealth interventions in relation to the clinical characteristics of patients.

**Objective:**

The objective of this study was to assess the access and use of, as well as experiences and interest in, new technologies using a survey of patients diagnosed with early psychosis compared with a survey of patients diagnosed with chronic psychotic disorders.

**Methods:**

We designed a structured questionnaire. This questionnaire was divided into five parts: (1) clinical and demographic information, (2) access and use of the internet, (3) use of the internet in relation to mental health, (4) experiences with technology, and (5) patients’ interest in eHealth services. In total, 105 patients were recruited from early psychosis units (n=65) and recovery units (n=40).

**Results:**

In this study, 84.8% (89/105) of the patients had access to the internet and 88.6% (93/105) owned an electronic internet device. In total, 71.3% (57/80) of patients who owned a mobile phone were interested in eHealth systems and 38.2% (37/97) reported negative experiences related to the internet usage. We observed differences between the groups in terms of device ownership (*P*=.02), the frequency of internet access (*P*<.001), the use of social media (*P*=.01), and seeking health information (*P*=.04); the differences were found to be higher in the early psychosis group. No differences were found between the groups in terms of the use of internet in relation to mental health, experiences and opinions about the internet, or interest in eHealth interventions (*P*=.43).

**Conclusions:**

The availability and use of technology for the participants in our survey were equivalent to those for the general population. The differences found between the groups in relation to the access or use of technology seemed to due to age-related factors. The use of technology involving mental health and the interest in eHealth interventions were mainly positive and equivalent between the groups. Accordingly, this group of patients is a potential target for the emerging eHealth interventions, regardless of their clinical status. However, 28.7% (23/80) of the studied patients rejected the use of internet interventions and 38.2% (37/97) had unpleasant experiences related to its usage; thus, more in-depth studies are needed to better define the profile of patients with psychosis who may benefit from eHealth treatments.

## Introduction

The relevance of early intervention (EI) in psychotic disorders in order to prevent the pathological development of the illness is well known [1]. However, some studies have shown that the current models of EI do not produce any different results in terms of efficacy or efficiency when compared with treatment as usual [2,3]. In this vein, technological developments could make a difference by adapting these traditional models of psychiatric and psychological health care to an electronic form, which would allow interactive and more personalized tracking of patients and Web-delivered therapy such as psychoeducational services or cognitive behavioral treatments [4]. These technological health interventions are known as eHealth [5]. The recent examples of these interventions that are being currently tested are Actissist [6], Prime [7], and SlowMo [8].

Nevertheless, before proceeding further in developing these eHealth interventions, it is important to better understand the relationship between patients with psychosis and technology resources. Psychotic disorders are characterized by their clinical heterogeneity [9]; thus, it is necessary to study if these eHealth interventions are equally accepted for all patients with psychosis, regardless of their demographics or clinical characteristics, especially if they are in an early psychosis (EP) condition or a chronic psychosis (CP) condition.

First, it is important to assess whether the access and use of technology are equivalent between EP and CP patients and whether the access and use are equivalent to those among the general population. Depp et al [10] conducted a survey of CP patients and found that these patients had substantial cognitive and functional deficits and that high punctuations in these impairments were related to the less use of technology. Moreover, in 2014, the National Alliance on Mental Illness (NAMI) [11] showed that 54% of American patients with schizophrenia owned a mobile phone compared with 64% of the general American population [12]; similar results have been shown in other studies [13]. However, recent studies have shown that these rates have changed and that the access of these patients to technology is similar to that of the general population at the moment [14-16].

Second, 80% of patients with psychosis are permitted to use internet resources in relation to their illness management [17]. Nevertheless, we could not find any study that investigated whether this use of technology is equivalent between EP and CP patients, who are usually more aged persons with more associated morbidities [10].

Third, despite the majority of patients who report positive feelings and experiences in response to the internet usage [4,18], there are some patients who experience anxiety or paranoid feelings while using this resource [18]. Moreover, some patients admitted that they had stopped taking medication on their own because of the information they read on the internet [17]. In relation to this, it is important to better understand the effect that technologies have on these patients and whether these experiences are similar between EP and CP patients.

Finally, there are several studies that have confirmed the interest of patients experiencing psychotic disorders in using the emerging eHealth systems to help them cope with their illness [4,14,18,19]. Specially, it has been found that 60%-75% of patients with psychosis would be interested in receiving information and feedback from their clinicians [19,20] and in contacting them in case of emergency [20]. However, there is a lack of studies that have assessed this interest in relation to the evolution of the disease (EP compared with CP). There are a few studies that have found some controversial results when studying this interest among individuals of different age groups. Some of these studies have suggested that younger patients would be more willing to endorse eHealth treatments [14,18], while others have suggested the opposite [21,22]. Consequently, it is necessary to study the variations in the interest in these services in relation to the evolution of the illness.

The main objective of this study was to assess the access and use of and experiences with technology in a survey of patients diagnosed with EP compared with a survey of patients diagnosed with CP disorder. In addition, we aimed to analyze the interest in these two groups regarding using an eHealth system and regarding the different tracking eHealth services suggested.

## Methods

### Measures and Design

The data were collected through a cross-sectional questionnaire that we designed for the purpose of this investigation. To elaborate this questionnaire, we reviewed studies about the use, access, and impact of technology on patients with psychosis. Based on these studies, we elaborated the survey, which is divided into five parts: the items for the first part, which aims to assess clinical and demographic information, and the items for the second part, which measures the access and use of the internet, mobile, and social media, were taken from the Spanish National Statistics Institute [23] survey and from studies by Trefflich et al [17] and Robotham et al [24]. In addition, the items for the third part of the questionnaire, which assesses the use of internet in relation to mental health, and the items for the fourth part, which measures experiences with technology and the effect of internet usage on patients’ health, were based on a survey of the NAMI [11] and on studies by Gay et al [18], Miller et al [25], and Borzekowski et al [26]. The last part of the survey, which rates the interest of the patients in using an eHealth app and their interest in different tracking and reminder services, was an originally developed section.

Once the instrument was made and prior to its use, a pilot study was conducted to check the acceptability and relevance of the measure. Overall, 14 representative patients participated; consequently, 3 ambiguous items were corrected in order to make them easier to understand for the patients, and 2 redundant items were removed.

The 10-minute, structured questionnaire (Multimedia Appendix 1) was completed face-to-face. Initially, the patients were informed about data extraction ethics and confidentiality following the information sheet (Multimedia Appendix 2); subsequently, the patients completed the questionnaire. All the patients signed the informed consent before participating in this survey. The survey was conducted from February to May 2017 and was approved by the Clinical Hospital of Valencia’s Ethics Committee.

### Sample and Recruitment

A total of 113 participants were eligible for inclusion. They met the following criteria: (1) diagnosis of a psychotic disorder according to the International Classification of Diseases, Tenth Revision [27]; (2) clinically stable; (3) outpatient from the first episode units at the Clinical Hospital of Valencia and from the Primary Care Centre Font of Sant Lluis in Valencia or outpatient from association for comprehensive care of the mental health patient or from aid association for mental health patients in the Valencia community recovery units; and (4) able to communicate in Spanish. Patients were excluded if they had severe cognitive impairments or did not complete the entire questionnaire.

### Data Analysis

We analyzed data using the statistical program IBM SPSS Statistics version 22. We excluded 8 patients from this analysis for not having totally filled the survey; due to this, data of 105 patients were considered for the analysis. The cohort was divided into two groups: the EP group, with a duration of illness of ≤72 months, and the CP group, with a duration of illness >72 months. This division was based on the fact that EP patients are treated in EP units until a maximum period of 72 months. Descriptive statistics (mean, standard deviation, frequencies, and percentages) were determined, and chi-square test and analysis of variance were performed in order to compare the differences between the EP and CP groups.

## Results

The data in Tables 1, 2, 3, and 4 are shown in the following order: First, the EP results are shown, followed by the CP results and the total results (which are the global results of the sample in each category). It is important to mention that the sample is not the same in every category due to the fact that some questions in the survey were exclusionary. If the patients did not fulfill the profile for one question, they did not have to complete the rest of the questions that were related to the first one. We have marked this condition in every table.

### Sample Characteristics

A total of 105 participants were enrolled in the study. Based on the duration of their illness, we assigned 65 patients to the EP group (≤72 months) and 40 patients to the CP group (>72 months). The mean age of the sample was 38.1 (SD 13) years; the patients were mostly male (76/105, 72.4%) and single (89/105, 84.8%) and had achieved a secondary level of education (compulsory schooling: 26/105, 24.8%; secondary education: 39/105, 37.1%).

We found significant differences between the two groups. EP patients were mostly in the first episode of psychosis (FEP), while CP patients were mostly diagnosed with schizophrenia. The duration (months) of illness was higher in the CP group. The patients in the EP group were younger and mostly employed, while those in the CP group were mostly unable to work or were not employed. There were no significant between-group differences in terms of gender, marital status, or the level of education. These clinical and sociodemographic characteristics are displayed in Table 1.

### Access and Use of the Internet, Mobile and Social Media

Of all the participants, 84.8% (89/105) had access to the internet in the 3 months prior to the study, and there was high electronic device availability in the survey (93/105, 88.6%). After the first two questions, 8 patients did not continue with completing the survey as they were considered “electronic excluded” patients because they were not using or had not used the internet sufficiently to consider their experience relevant for the aim of this study. From that moment on, the total sample consisted of 97 patients (EP, n=63; CP, n=34).

Differences between the groups (Table 2) were found in terms of electronic device availability (*χ*^2^_5_=13.8, *P*=.02), the frequency of access to the internet (*χ*^2^_2_=31.8, *P*<.001), and the use of social media (*χ*^2^_4_=13.9, *P*=.01). Electronic device availability was higher in the EP group (63/65, 97%) than in the CP group (30/40, 75%), and while 81% (51/63) patients in the EP group had daily access to internet, 52.9% (8/34) of the patients in the CP group had only weekly access. However, no differences were observed in terms of the type of device used to access (*χ*^2^_2_=5.6, *P*=.06), mobile ownership (*χ*^2^_5_=10.2, *P*=.07), or the most used functions of the mobile phone, which were calls (74/88, 84.1%; *χ*^2^_1_=0.7, *P*=.41) and texting or WhatsApp (72/88, 83.8%; *χ*^2^_1_=0.4, *P*=.51) for both groups. Social media ownership was higher in the EP group (51/63, 81%) than in the CP group (15/34, 44.1%); however, Facebook was the most used social media site in both the groups (47/66, 72.3%; *χ*^2^_1_=1.6, *P*=.21), and patients’ main goal in using this social media platform was to communicate with people (55/66, 83.3%; *χ*^2^_1_=1.4, *P*=.24).

### Internet and Mental Health

Internet is a resource that 61.9% (60/97) of the patients used to seek information about health. EP patients (45/63, 71.4%) used this resource to a greater extent than CP patients (15/34, 44.1%; *χ*^2^_5_=11.5, *P*=.04). The most wanted information was regarding symptoms (47/60, 78.3%) or diagnosis (40/60, 66.7%), which was more sought after by CP patients (14/15, 93.3%) than by EP patients (26/45, 57.8%; *χ*^2^_1_=6.4, *P*=.01). Of all the patients, 37.1% (36/97) stated that the internet was their first resource for seeking health information, whereas 58.8% (57/97) consulted clinical services as a first option.

In relation to the feelings that the use of internet provided to the patients, we found that 60.9% (59/97) felt socially linked when using internet and that 78.4% (76/97) felt informed. However, 22.7% (22/97) of the patients felt frustrated or anxious in relation to the internet and 19.6% (19/97) felt suspicious or paranoid.

**Table 1:** Demographic and clinical characteristics.

Characteristics		Early psychosis (N=65)	Chronic psychosis (N=40)	Total (N=105)	*P* value (*χ*^2^^a^ or *t*^b^, *df*^c^)
**Diagnosis, n (%)**					**<.001 (61.9^a^, 7)**
	Schizophrenia	9 (13.8)	29 (72.5)	38 (36.2)	
	First episode of psychosis	44 (67.7)	0 (0.0)	44 (41.9)	
	Other psychotic disorder^d^	12 (18.5)	18 (27.5)	23 (21.9)	
Duration of Illness (months), mean (SD)		28.8 (21.3)	253.3 (115)	114.3 (131.3)	<.001 (235.9^b^, 1)
Age (years), mean (SD)		32.9 (11.8)	46.6 (10.3)	38.1 (13)	<.001 (−6.1^b^, 103)
**Gender, n (%)**					**.38 (.8^a^, 1)**
	Female	16 (24.6)	13 (32.5)	29 (27.6)	
	Male	49 (75.4)	27 (67.5)	76 (72.4)	
**Marital status, n (%)**					**.07 (7.2^a^, 3)**
	Single	56 (86.2)	33 (82.5)	89 (84.8)	
	Married	7 (10.8)	1 (2.50)	8 (7.6)	
	Widowed	0 (0)	1 (2.50)	1 (1.0)	
	Divorced	2 (3.1)	5 (12.5)	7 (6.7)	
**Education, n (%)**					**.43 (3.8^a^, 4)**
	Primary school	11 (16.9)	8 (20)	19 (18.1)	
	Compulsory schooling^e^	17 (26.2)	9 (22.5)	26 (24.8)	
	Secondary education	22 (33.8)	17 (42.5)	39 (37.1)	
	University degree	15 (23.1)	6 (15.0)	21 (20.0)	
**Employment status, n (%)**					**<.001 (27.7^a^, 6)**
	Employed	18 (27.7)	4 (10.0)	22 (21.0)	
	Not employed	16 (24.6)	11 (27.5)	27 (25.7)	
	Student	16 (24.6)	1 (2.5)	17 (16.2)	
	Unable to work	9 (13.8)	18 (45.0)	27 (25.7)	
	Others	6 (9.3)	6 (15.0)	12 (11.5)	

^a^Chi-square (χ^2^) values.

^b^Student *t* values.

^c^*df*: degrees of freedom.

^d^Referring more than one psychotic episode or a specific disorder (bipolar, schizophreniform, schizoaffective, major depression, personality disorder).

^e^Until the age of 16 years.

**Table 2:** Access to and use of the internet, mobile, and social media.

Access and use of technology		Early psychosis (N=65), n (%)	Chronic psychosis (N=40), n (%)	Total (N=105), n (%)	*P* value (*χ*^2^, *df*^a^)
**Internet access (last 3 months)**		**N=65**	**N=40**	**N=105**	**.05 (7.5, 3)**
	Yes	59 (90.8)	30 (75)	89 (84.8)	
	No	6 (9.2)	10 (25)	16 (15.2)	
**Electronic device availability**		**N=65**	**N=40**	**N=105**	**.02 (13.8, 5)**
	Yes	63 (97)	30 (75)	93 (88.6)	
	No	2 (3)	10 (25)	7 (11.4)	
**Device type**		**N=63**	**N=30**	**N=93**	**.06 (5.6, 2)**
	Computer	16 (25.4)	13 (43.3)	29 (31.2)	
	Mobile	47 (74.6)	16 (53.3)	63 (67.7)	
	Tablet	0 (0)	1 (3.3)	1 (1.1)	
**Frequency of internet access**		**N=63**	**N=34**	**N=97**	**lt;.001 (31.8, 2)**
	Daily	51 (81)	11 (32.4)	62 (63.9)	
	Weekly	3 (4.8)	18 (52.9)	21 (21.6)	
	Less than once a week	9 (14.3)	5 (14.7)	14 (14.4)	
**Mobile ownership**		**N=63**	**N=34**	**N=97**	**.07 (10.2, 5)**
	Yes, cell phone	7 (11.1)	6 (17.6)	13 (13.4)	
	Yes, mobile phone	54 (85.7)	21 (61.8)	75 (77.3)	
	No	2 (3.2)	7 (20.6)	9 (9.3)	
**Mobile use^b^**		**N=61**	**N=27**	**N=88**	
	Calls	50 (82)	24 (88.9)	74 (84.1)	.41 (.7, 1)
	Texting or WhatsApp	51 (83.6)	21 (77.8)	72 (83.8)	.51 (.4, 1)
**Social media ownership**		**N=63**	**N=34**	**N=97**	**.01 (13.9, 4)**
	Yes	51 (81)	15 (44.1)	66 (68)	
	No	12 (19)	19 (55.9)	31 (32)	
**Social media site^b^**		**N=51**	**N=15**	**N=66**	
	Facebook	351 (68.6)	12 (80)	47 (72.3)	.21 (1.6, 1)
	WhatsApp groups	34 (66.7)	12 (80)	46 (69.7)	.32 (.9, 1)
**Social media use^b^**		**N=51**	**N=15**	**N=66**	
	To communicate with people	44 (86.3)	11 (73.3)	55 (83.3)	.24 (1.4, 1)
	To stay informed	35 (68.6)	9 (64.3)	44 (67.7)	.76 (.1, 1)

^a^*df*: degrees of freedom.

^b^Sample reduction because of a previous exclusionary question.

**Table 3:** Internet and mental health.

Experiences and opinions about internet		Early psychosis (N=63), n (%)	Chronic psychosis (N=34), n (%)	Total (N=97), n (%)	*P* value (*χ*^2^, *df*^a^)
**Internet used to seek health information**		**N=63**	**N=34**	**N=97**	**.04 (11.5, 5)**
	Yes	45 (71)	15 (44)	60 (62)	
	No	18 (29)	19 (56)	37 (38)	
**Most sought after health information^b^**		**N=45**	**N=15**	**N=60**	
	Symptoms	36 (80)	11 (73)	47 (78)	.59 (.3, 1)
	Diagnosis	26 (58)	14 (93)	40 (67)	.01 (6.4, 1)
**Internet: first information resource**		**N=63**	**N=34**	**N=97**	.13 (5.7, 3)
	Agree	27 (43)	9 (27)	36 (37)	
	Disagree	36 (57)	25 (74)	61 (63)	
**Agreement on internet feelings^c^**		**N=63**	**N=34**	**N=97**	
	Socially linked	28 (60)	21 (62)	59 (61)	.93 (.9, 5)
	Informed	53 (84)	23 (68)	76 (78)	.24 (5.5, 4)
	Frustrated or Anxious	11 (18)	11 (32)	22 (23)	.08 (8.3, 4)
	Suspicious or Paranoid	13 (21)	6 (18)	19 (20)	.46 (3.6, 4)
**Agreement on internet experiences^c^**		**N=63**	**N=34**	**N=97**	
	Internet as a benefit for mental health	27 (43)	18 (53)	45 (46)	.23 (5.6, 4)
	Unpleasant experiences related to internet usage	25 (40)	12 (35)	37 (38)	.92 (.9, 4)
	Stopped taking medication because of internet information	4 (6)	4 (12)	8 (8)	.84 (1.4, 4)
	Relapse related to internet usage	20 (32)	4 (12)	24 (25)	.17 (6.4, 4)
	Excessive time spent on internet	20 (32)	6 (18)	26 (27)	.25 (5.4, 4)
	Internet increases social isolation	12 (19)	5 (15)	17 (16)	.69 (2.3, 4)

^a^*df*: degrees of freedom.

^b^Sample reduction because of a previous exclusionary question.

^c^Sum of individual scores of “Strongly agreed” and “Somewhat agreed” in each factor.

Regarding experiences related to internet usage, we found that 46.4% (45/97) of the patients thought that the internet is beneficial to their mental health, while 38.2% (37/97) had unpleasant experiences related to its usage, and 24.8% (24/97) patients had experienced relapses perceived as directly related to internet usage. Moreover, 8.3% (8/97) patients had stopped taking medication on their own because of the information they read on the internet. Excessive time on the internet was a concern for 26.8% (26/97) of the patients and 16.2% (17/97) thought that internet increases social isolation. As displayed in Table 3, we could not find any significant between-group differences in terms of the feelings about the internet or experiences related to its usage.

### Interest in eHealth Systems (Mobile Phone App)

This part of the survey was completed only by patients who owned a mobile phone. For this reason, the sample size was reduced to 80 patients (EP, n=59; CP, n=21). Of all the patients, 71.3% (57/80) were interested in owning an eHealth app, with no significant differences observed between the EP and CP groups (*χ*^2^_4_=3.9; *P*=.43); furthermore, no significant differences were observed in terms of age of the sample (*F*_1_=.08, *P*=.93). The reason for not being interested was “I do not think I will benefit from it” (14/23, 60.9%) or “I have enough information” (6/23, 26.1%).

The services that were perceived as the most interesting were as follows: clinician contact alarm (60/80, 75.1%) and a reminder for clinical appointments (58/80, 72.6%). Mood, mental health, and side effect tracking were perceived as equally interesting (51/80, 63.8%), while the least interesting function for the patients was the reminder to take medication (41/80, 51.3%). As shown in Table 4, no significant differences were found between the groups in terms of their interest in any of the services suggested. Furthermore, no significant differences were found regarding the age of the sample and interest in mood and mental health service (*F*_1_=1.31, *P*=.27), interest in side effect tracking (*F*_1_=1.44, *P*=.24), reminder for clinical appointments (*F*_1_=.99, *P*=.37), reminder to take medication (*F*_1_=.2.35, *P*=.11), and clinician contact alarm (*F*_1_=.47, *P*=.63).

**Table 4:** Interest in eHealth systems (app).

Opinions about eHealth app services			Early psychosis (N=59), n (%)	Chronic psychosis (N=21), n (%)	Total (N=80), n (%)	*P* value (*χ*^2^, *df*^a^)
**App interest**						**.43 (3.9, 4)**
	Yes		42 (71.2)	15 (71.4)	57 (71.3)	
	**No**		**17 (28.8)**	**6 (28.6)**	**23 (28.7)**	
		I do not think I will benefit from it^b^	11 (64.7)^c^	3 (50)^d^	14 (60.9)^e^	
		I have enough information^b^	5 (29.4)^c^	1 (16.7)^d^	6 (26.1)^e^	
		Others^b^	1 (5.9)^c^	2 (33.3)^d^	3 (13)^e^	
**App services**						
	**Mood and mental health tracking**					**.77 (1.8, 4)**
		Interested^f^	38 (64.4)	13 (61.9)	51 (63.8)	
		Indifferent	5 (8.5)	3 (14.3)	8 (10)	
		Not interested^g^	16 (27.1)	5 (23.8)	21 (26.3)	
	**Side effect tracking**					**.39 (4.1, 4)**
		Interested^f^	40 (67.8)	11 (52.3)	51 (63.8)	
		Indifferent	4 (6.8)	4 (19)	8 (10)	
		Not interested^g^	15 (25.4)	6 (28.5)	21 (26.3)	
	**Reminder of clinical appointments**					**.82 (1.5, 4)**
		Interested^f^	41 (69.5)	17 (80.9)	58 (72.6)	
		Indifferent	4 (6.8)	1 (4.8)	5 (6.3)	
		Not interested^g^	14 (23.7)	3 (14.3)	17 (21.3)	
	**Reminder to take medication**					**.32 (4.7, 4)**
		Interested^f^	29 (49.1)	10 (57.1)	41 (51.3)	
		Indifferent	11 (18.6)	2 (9.5)	13 (16.3)	
		Not interested^g^	19 (32.2)	7 (33.3)	26 (32.6)	
	**Clinician contact alarm**					**.12 (7.3,4)**
		Interested^f^	44 (74.5)	16 (76.2)	60 (75.1)	
		Indifferent	4 (6.8)	—	4 (5)	
		Not interested^g^	11 (18.6)	5 (23.8)	16 (20)	

^a^*df*: degrees of freedom.

^b^Sample reduction because of a previous exclusionary question.

^c^N=17.

^d^N=6.

^e^N=23.

^f^Sum of individual scores of “Very interested” and “Somewhat interested” in each factor.

^g^Sum of individual scores of “Not very interested” and “Not at all interested” in each factor.

## Discussion

### Access and Use of Technology

The rates of accessibility and usability of the internet, mobile, and social media in our surveyed sample were high and very similar to the rates we found in the general Spanish population [23]. These results contradict the lower rates obtained by the NAMI study in 2014 [11] and are more similar to the results of recent studies [14-16,18,28], which found that the access and use of technology in patients diagnosed with psychotic disorders are equivalent to those in the general population. The differences between the two comparison groups in this study suggested that the access and use are not equivalent between EP and CP patients. As we found, CP patients had less electronic device availability (CP: 30/40, 75%; EP: 63/65, 97%) as well as lower rates of daily access to the internet (CP: 11/34, 32.4%; EP: 51/63, 81%) and use of social media (CP: 15/34, 44.1%; FEP: 51/63, 81%) than EP patients. However, these differences were not only found in previous studies on patients with psychosis [14,17,24,29] but also found in studies on the general Spanish population [23,30]. All these studies agreed that younger patients (18-34 years) have the highest rates of access and use of technology and that these rates start to decrease with the increasing age. In relation to this, we suggest that the differences found between EP and CP patients might be more related to the fact that EP patients were younger than CP patients (*P*<.001) than to a pathologically related issue.

### Use of the Internet Related to Mental Health

In accordance with previous studies [17,23,28,31], the internet is a resource that both patients and the general population use in order to seek information about health. Moreover, nearly 40% (39/97) of our patients admitted that the internet is their first source of health information. However, in accordance with previous studies [17], EP patients used this resource to a greater extent than CP patients (EP: 45/63, 71.4%; CP: 15/34, 44.1%; *P*=.04). Nevertheless, it is important to note that nearly 56% (19/36) of CP patients and 29% (18/63) of EP patients did not use the internet to seek health information and that nearly 63% (61/97) of patients did not regard internet as their first source of information. These results suggest that despite the fact that the internet is an accessible and quick resource to obtain information [28,32], patients still rely on clinicians as their first source of health information.

### Experiences and Opinions About the Internet

In line with previous studies [4,18,32], between 60.9% (59/97) and 78.4% (76/97)of patients reported positive experiences related to the internet usage. However, 22.7% (22/97) of the patients felt frustrated or anxious in relation to the internet, and 19.6% (19/97) felt suspicious or paranoid. Moreover, 38.2% (37/97) of the patients had had unpleasant experiences related to internet usage, 24.8% (24/97) had experienced relapses perceived as directly related to its usage, and 8.3% (8/97) of the sample had stopped taking their medication on their own decision because of the information that was read on the internet. It should be noted that despite the fact that the access and use of technology were found to be higher in the EP group, there were no between-group differences in relation to their experiences of or opinions on internet usage. However, these negative experiences have been found in previous studies [18,29,32], and they suggest that although internet could be a great resource to improve the empowerment of the patients in the management of their illness [32] or as an entertainment resource [18], it could also be a source of stress by causing anxious or paranoid feelings [18,29,32]. It is important to mention that 50%-56% of the general Spanish population agrees with “being worried about internet, social media, and government use of personal information given on the internet” [33]; in accordance with this, we suggest that new technologies are a source of information that could be interpreted as a false alarm signal that may trigger paranoid symptoms. However, we could not find any studies concerning this issue.

Moreover, although 60.9% (59/97) of the patients felt socially linked when using the internet, 26.8% (26/97) admitted to spending excessive time on it and 16.2% (17/97) thought that internet increases social isolation. This enhancement of social isolation has also been reported in studies in the general population [34]. In accordance with previous studies, social isolation is a risk factor for psychosis [35], and it is one of the key relapse factors following the FEP [36].

### Interest in eHealth Systems (Mobile Phone App)

Consistent with previous studies [4,14,19], the interest in owning an eHealth system (mobile phone app) in our sample was high (57/80, 71.3%), with no differences observed between the two comparison groups. Moreover, we could not find any significant differences between the groups in terms of their interest in the different eHealth services suggested or when comparing the age of the sample. This result has been found in a systematic review of previous acceptance studies [19], which concluded that there is no difference between clinical and demographic characteristics and the acceptance of eHealth interventions. In line with this, the high acceptance of eHealth interventions in our sample could be regarded as a potential confirmation that patients with psychotic disorders are a good target for these emerging interventions, with no differences related to the length of the illness.

However, although the differences were not statistically significant, on comparing both groups, we found that the percentages of interest were higher in the CP group than in the EP group regarding “reminder services” (clinical appointments and taking medication). In a previous study [21], it was found that the older the patients were, the more reminders they would select. In line with that study, we suggest that CP patients, being more aged and impaired than EP patients, as shown in Table 1 and in previous studies [10], could regard reminder services as a helpful tool to manage their illness, whereas the EP group, being younger and having better social support and less associated impairments, would not regard this service as useful.

On the other hand, EP patients found the “tracking services” (mood, mental health and, side effects) more interesting than CP patients. In a systematic review of previous publications [19], it was found that the interest of patients in receiving psychoeducative and symptom information increased to 90% in the EP sample. According to this finding, we speculated that the EP group would consider “tracking services” more interesting due to their more recent diagnosis and need to better understand their illness, whereas the more experienced CP patients would not consider this service useful.

However, as noted before, there were no significant differences between the groups; thus, initially, patients in both the groups (EP and CP) would be interested in any service in an equivalent way regardless of their age.

Finally, it is important to mention that the most interesting service for the patients was the “contact alarm to the clinicians in case of emergency” (60/80, 75.1%); the interest shown by both groups was nearly the same (EP: 44/59, 74.5%; CP: 16/21, 76.2%). This service must be a priority in eHealth developments. Patients are asking for more personalized, interactive, and closer clinical attention [14,19,20], which could lead to a greater improvement in psychosis EI [4]. However, as noticed in previous studies [37,38], regarding the clinical implications associated to these interventions, it is highly important to design these feedback systems taking into consideration the clinicians’ perspective to not overwhelm their capacities to respond to this systems.

### Limitations

This study has some limitations. First, we cannot generalize the results to a broader population of individuals with psychotic disorders. We could not conduct a randomized selection of the sample; therefore, it was selected for the purpose of the aim of this study. Moreover, the small sample size (N=105) and the fact that 72.4% (46/105) of the patients were males with a mean age of 38.1 (SD 13) years caused our sample to not be representative of the demographic distribution of individuals with psychosis. In addition, some demographic information, such as the ethnicity of the sample was not collected. However, it is important to note that most of our results are consistent with those of previous publications; thus, we could infer that in a larger, randomized sample, the results would be similar to the ones we obtained in this study.

Second, the data were obtained from a questionnaire designed for the purpose of this investigation. Even though it was based on a previous review of publications and we conducted a pilot study to test its validity, our survey was not a standardized or a properly validated instrument for individuals with psychotic disorders. The quality of data obtained was affected for this reason. Moreover, most of the items in the survey measured nominal information, which hampered the performance of more complex statistical analyses. In relation to this, some items measured opinions or patients’ perceptions, and we did not include an open text-box in order to better understand the responses given by the patients to these items.

Finally, regarding items of the final section of the questionnaire, since eHealth services are rapidly progressing, future updates of these items would be needed.

### Implications and Orientations for Future Research

This study highlighted the viability and relatively high acceptability of eHealth interventions in a sample of patients diagnosed with psychotic disorders. However, some disregarded issues must guide future investigations in the area of eHealth and psychotic disorders.

First, although the findings of this study that is related to the access and usability of new technologies in patients diagnosed with psychotic disorders are very similar to the data obtained in the previous studies conducted in patients with psychosis [14,17,18,24,31] and in studies conducted in the Spanish general population [23,33], larger studies are needed to generalize our results, based on a small sample, to a broader patient population with psychosis in Spain to confirm that they are a good target for eHealth interventions.

Second, our results showed that there is a widespread use of internet to obtain information about health, not just by patients diagnosed with psychotic disorders but also by the general population [23]. However, we would like to highlight the substantial negative experiences related to internet usage that we found in our sample. Due to the great extent of internet usage in our society, we believe that further studies focusing on how internet usage affects patients are needed to understand the effect that this resource has on these patients and to study its role as a risk factor for psychosis.

Finally, we did not find any differences between the patient groups in terms of interest in eHealth services, allowing us to conclude that regardless of the demographic or clinical characteristics of patients, they would be equally interested in these interventions. However, in every category measured, we found 20%-30% of patients who were systematically “not interested” in the interventions suggested. As it has been shown in previous studies, personality can affect internet and mobile phone use [39,40]. In accordance with this, it would be interesting to replicate this study with a larger sample and to include specific measures of personality, interest, and patients’ expectations because we believe that it would not be possible to achieve any promising results with the use of technology advances if the patients do not feel encouraged and motivated to use these resources. This is the reason why future investigations must focus on better understanding the patients’ point of view to truly achieve a personalized measure of the patients’ health status.

This study is the first approach to such patients’ perspective. We aimed to describe patients’ current situation in terms of the availability of technology and the experiences and opinions related to its usage. However, further studies are needed.

## Appendix

Multimedia Appendix 1 Survey instrument.

Multimedia Appendix 2 Ethics and confidentiality. Information sheet for the patient.

## Abbreviations

CP: chronic psychosis
EI: early intervention
EP: early psychosis
FEP: first episode of psychosis
NAMI: National Alliance on Mental Illness
