# Supplementary material for: Differences in the Use and Opinions About New eHealth Technologies Among Patients With Psychosis: Structured Questionnaire
Source: JMIR Ment Health. 2018 Jul 25;5(3):e51. doi: 10.2196/mental.9950 (PMC6083047; doi:10.2196/mental.9950)
Supplement: Multimedia Appendix 1 [file mental_v5i3e51_app1.pdf]

SCHOOL OF MEDICINE, UNIVERSITY OF VALENCIA

MENTAL HEALTH AND NEW TECHNOLOGIES SURVEY

The aim of this survey is to assess the access, use, experiences and opinions related to new technologies (internet, mobile phones, social media) in patients from Mental Health Services in Valencia. Moreover, we aim to assess the interest of these patients in different electronic health services.

Information obtained from this survey will be used for research or academic purposes. Your responses will be confidential, and only the researchers involved in this study will see this information.

Your cooperation is essential for us, so please read the instructions given below carefully and complete the entire questionnaire.

RESPONSES WILL BE CONFIDENTIAL AND ANONYMOUS.

THANK YOU FOR YOUR COOPERATION.

QUESTIONNAIRE

INSTRUCTIONS:

The following items have different possible answers; please mark the most appropriate response. Unless it has been specifically indicated, do not mark more than **<u>one response</u>** for each item. Be as sincere as possible; there are no good or bad responses.

1. DEMOGRAPHIC DATA:

Diagnosis:
Age:
Length of illness:

- *Gender:*
  - ☐ Female
  - ☐ Male

- *Marital status:*
  - ☐ Single
  - ☐ Married
  - ☐ Widowed
  - ☐ Divorced/Separated

- *Highest level of education achieved:*
  - ☐ Primary school or less
  - ☐ Compulsory schooling
  - ☐ Secondary education
  - ☐ University degree or more

- *Employment status:*
  - ☐ Employed
  - ☐ Not employed
  - ☐ Student
  - ☐ Unable to work
  - ☐ Other

2. TECHNOLOGY ACCESS:

NOTE: When we ask about the **internet** without any specifications, we are asking about access to the internet by using <u>any</u> kind of <u>device</u> (computer, laptop, mobile phone, tablet) or the use of any <u>internet platform</u> (websites, apps, social media, etc.).

- ***Have you accessed the internet in the last 3 months?***

☐ YES

☐ NO………………………… Argue your response:

☐ I do not have access to devices (mobile, computer, etc.) or services (library, cibers, etc.) to use the internet.
☐ I do not know how to access the internet.
☐ I think the internet is dangerous to my security and/or privacy.
☐ Other (write your own argument)

- ***Do you own an electronic device (mobile, computer, laptop, tablet, etc.) to access the internet?***

☐ YES……..:

☐ I own a personal device
☐ I share a device with a relative or someone I live with.
.

☐ NO………………………… Argue your response:

☐ I do not need the internet (it is not useful, or it is not interesting to me).
☐ The cost is very expensive (the cost of the device or internet).
☐ I do not know how to use it.
☐ Electronic devices are dangerous to my security and/or privacy.
☐ Other (write your own argument)

- ***If you have answered YES to the last question, mark the type of electronic device you own to access the internet. If you own more than one, mark the device you use most frequently:***

☐ Computer or laptop
☐ Mobile phone
☐ Tablet
☐ Other:

3. USE OF TECHNOLOGY:

NOTE: *If you have answered* ***NO*** *to the first of the items, please finish the questionnaire.*

☐ YES

☐ NO............................ Argue your response:

- ☐ I do not need the internet (it is not useful, or it is not interesting to me).
- ☐ The cost is very expensive (the cost of the device or internet).
- ☐ I do not know how to use it.
- ☐ Electronic devices are dangerous to my security and/or privacy.
- ☐ Other (write your own argument)

- ***How often do you access to internet?***

☐ Daily, at least 5 times a week

☐ Weekly, but not everyday

☐ Monthly, less than once a week

- ***Do you own a mobile phone?***

☐ YES............................ Cross the type of phone:

- ☐ Traditional mobile phone
- ☐ Smartphone (access to internet)

☐ NO............................ Argue your response:

- ☐ I do not need a mobile phone (it is not useful, or it is not interesting to me).
- ☐ The cost is very expensive (the cost of the device or internet).
- ☐ I do not know how to use it.
- ☐ Mobile phones are dangerous to my security and/or privacy.
- ☐ Other (write your own argument)

- ***Please, mark the mobile phone services you have used in the last 3 months. Mark all that apply:***

☐ Make/receive calls
☐ Send messages (WhatsApp, SMS, etc.)

☐ Surfing the internet
☐ Access to social media
☐ Take/share photos

- ***Have you ever used a social media platform (Facebook, Instagram, Twitter, etc.)?***

☐ YES

☐ NO............................ Argue your response and move to PART 4:

  ☐ Lack of access to the internet.
  ☐ I do not think social media are interesting.
  ☐ I do not know how to use it.
  ☐ Social media are dangerous for my security and/or privacy.
  ☐ Others (write your own argument)

- ***Which social media platforms do you use the most? Mark all that apply:***

☐ Facebook

☐ Twitter

☐ Instagram

☐ LinkedIn

☐ WhatsApp Groups

☐ Others

- ***Why do you use social media platforms? Mark all that apply:***

☐ To stay in touch with friends and people I know (to watch and comment on their photos, to chat with them, etc.)
☐ To stay up to date with the news
☐ To stay in touch with my family
☐ To work or employment-related issues
☐ To find a couple or a date

☐ Other

4. USE OF TECHNOLOGY IN RELATION TO MENTAL HEALTH:

- ***Have you ever sought information about mental health on the internet?***

  ☐ YES

  ☐ NO………………………. Argue your response:

  ☐ I do not know how to seek this information on the internet.
  ☐ I do not have availability of devices or services to seek information on internet.
  ☐ Information given by the clinicians was enough.
  ☐ Seeking this information on the internet is dangerous to my security and/or privacy.
  ☐ Other (write your own argument)

- ***In case you answered YES to the last item:***

  - ***Which kind of information did you seek? Mark all that apply:***

    ☐ Diagnosis
    ☐ Symptoms
    ☐ Medication
    ☐ Treatments
    ☐ Medication side-effects
    ☐ Other

  - ***Is the internet your first resource for obtaining information about mental health?***

    ☐ YES

    ☐ NO………………:

    ☐ My first sources of information are medical services (psychiatrist, psychologist, doctors, etc.).

- [ ] My first source of information is my family or my friends.
- [ ] I use other sources of information.

- ***How often do you use the internet in relation to the following issues of your mental health?***

	Daily	Weekly	Monthly	Never
Seeking information: symptoms, medication, side-effects, etc.	☐	☐	☐	☐
Seeking new treatments or therapeutic techniques	☐	☐	☐	☐
Use of music and other resources to help cope with auditory hallucinations	☐	☐	☐	☐
To participate in support groups	☐	☐	☐	☐

5. EXPERIENCES AND OPINIONS RELATED TO INTERNET USAGE:

- ***Indicate your agreement or disagreement with how the internet makes you feel by crossing off your response on the scale:***

	Strongly agree	Somewhat agree	Neutral	Somewhat disagree	Strongly disagree

Socially linked	☐	☐	☐	☐	☐
Informed	☐	☐	☐	☐	☐
Entertained	☐	☐	☐	☐	☐
Frustrated/ Anxious	☐	☐	☐	☐	☐
Suspicious/ Paranoid	☐	☐	☐	☐	☐

- ***Indicate your agreement or disagreement with the following statements by crossing off your response on the scale:***

	Strongly agree	Somewhat agree	Neutral	Somewhat disagree	Strongly disagree
As a whole, I think the internet is beneficial to my mental health.	☐	☐	☐	☐	☐
I had unpleasant experiences related to internet usage.	☐	☐	☐	☐	☐
I stopped taking medication because of information I read on the internet.	☐	☐	☐	☐	☐

	Strongly agree	Somewhat agree	Neutral	Somewhat disagree	Strongly disagree
I had relapses related to internet usage.	☐	☐	☐	☐	☐
I spend too much time on the internet.	☐	☐	☐	☐	☐
The internet has increased my social	☐	☐	☐	☐	☐

isolation.

NOTE: Complete this part ONLY if you own a SMARTPHONE (mobile phone with internet).

6. FUTURE RESEARCH:

- **Would you be interested in owning a smartphone app to help you cope with your illness and to improve your mental health?**

  ☐ YES

  ☐ NO………………………… Argue your response:

  - ☐ I have enough information and support.
  - ☐ For me, it is difficult to use a smartphone app.
  - ☐ I do not think I am going to take any benefit from its usage.
  - ☐ Other

- ***Indicate your level of interest in the following app services by crossing off your response on the scale:***

	Very interested	Somewhat interested	Neutral	Not very interested	Not at all interested
Mood and mental health tracking	☐	☐	☐	☐	☐
Medication side-effects tracking	☐	☐	☐	☐	☐

Reminder of clinical appointments	☐	☐	☐	☐	☐
Reminder to take medication	☐	☐	☐	☐	☐
Clinicians contact alarm in case of emergency	☐	☐	☐	☐	☐
