# Supplementary material for: Differences in the Use and Opinions About New eHealth Technologies Among Patients With Psychosis: Structured Questionnaire
Source: JMIR Ment Health. 2018 Jul 25;5(3):e51. doi: 10.2196/mental.9950 (PMC6083047; doi:10.2196/mental.9950)
Supplement: Multimedia Appendix 2 [file mental_v5i3e51_app2.pdf]

Multimedia Appendix 2

Ethics and confidentiality. Information sheet for the patient.

You are being invited to take part in a research study named “Mental health and new technologies”. This study is being conducted by Lucia Bonet Mora, a researcher from the School of Medicine in Valencia. Before you decide to take part in this study, it is important for you to understand why this research is being done and what it will involve. Please take time to understand the following information carefully and to decide whether or not you wish to take part.

1. **What is the purpose of the study?**
   The aim of this study is to assess the access, use and experiences with technology in a survey of patients diagnosed with first-episode psychosis compared to a survey of patients diagnosed with chronic psychotic disorders. In addition, we aim to analyse the interest between these two groups in different e-Health services.

2. **Why have you been chosen?**
   You have been chosen because you have been diagnosed with a psychotic disorder.

3. **What do you have to do? What will happen to you if you decide to participate?**
   Participation in this study will not affect any of your functioning areas, and it will not affect your usual medical care.
   Once you have finished your current appointment with the clinician, you will have to complete a ten-minute survey. After this, your results will be analysed in terms of the investigative purpose.
4. **What are the possible risks for taking part in this study?**
   There is no risk for participating in this study, and there is not any inconvenience or discomfort associated with participation.

5. **What are the possible benefits of participating?**
   This study aims to describe the profile of a patient that would be a good target for owning a smartphone app that helps to manage the psychosis illness. The result from this study will not have a direct impact on your health, but they will help us to know your interest and preferences in order to develop this app.

In the future, when this app is developed by following the information we collect in this survey, you may use this app to improve the quality of the health care attention you are receiving.

6. **What will happen to me if I decide not to take part?**
   Participation in this study is voluntary. It is completely up to you to decide whether or not to participate. If you decide not to participate, it will not affect the treatment you are receiving now or in the future. Moreover, if you decide to take part, you are still free to withdraw at any time and without giving a reason. It will not affect your relationship with the clinicians caring for your health.

7. **Who should I contact if I have concerns about this study?**
   It is important that you feel free to tell your clinician or the researchers any concern or complaint about this study. However, if you need further information, please contact the main researcher, Lucia Bonet Mora at 600745550.

8. **Confidentiality:**
   If you consent to take part in this study, your medical or personal information may be inspected by the researchers involved in the study. However, any identifiable information collected will remain strictly confidential.
   If the results from this survey are published in scientific journals, any identifiable information collected about you will be removed so that you cannot be recognized from it.

Thank you for taking the time to consider this study.
